# Paramedic assessment of carotid artery pulsation using pre-recorded ultrasound videos: a comparative analysis of three ultrasound modes

**DOI:** 10.1016/j.resplu.2025.101028

**Published:** 2025-07-11

**Authors:** C. Gaik, H. Wulf, B. Vojnar

**Affiliations:** Department of Anesthesiology and Intensive Care Medicine, University Hospital Giessen and Marburg, Campus Marburg and Philipps University of Marburg, Germany

**Keywords:** Cardiac arrest, Carotid ultrasound, Emergency medical service, Paramedics, POCUS, Pulse check

## Abstract

**Background:**

This cross-sectional study aimed to determine whether paramedics with limited or no prior ultrasound experience can consistently identify the presence or absence of common carotid artery (CCA) pulsation, as a potential alternative to manual pulse checks during cardiopulmonary resuscitation.

**Methods:**

Following a six-minute instructional video, paramedics assessed short pre-recorded ultrasound videos of the CCA acquired in B-mode, M−mode, and Color Doppler. Each of the 93 participants viewed 24 randomized 10-second videos and classified each as showing pulsation or no pulsation. To replicate clinical conditions where manual pulse checks may fail − such as post-resuscitation − videos were recorded during two distinct phases of cardiac surgery: (1) under controlled hypotension and (2) during complete circulatory standstill following aortic clamping.

**Results:**

A total of 2232 assessments were analyzed. M−mode: Participants correctly identified CCA pulsation in 95 % (265/279) of assessments. In videos without CCA pulsation, the correct classification of ‘no pulsation present’ was achieved in 97 % (270/279). B-mode: The presence of CCA pulsation was correctly identified in 78 % (218/279), whereas in 22 % (61/279) of cases, participants incorrectly categorized the video as ‘no pulsation present’ despite pulsation being present. Conversely, the absence of CCA pulsation was accurately detected in 98 % (635/651) of cases. Color Doppler: CCA pulsation was correctly identified in 99 % (551/558) of assessments. Similarly, in videos without CCA pulsation, participants correctly classified 96 % (185/186) as ‘no pulsation present’.

**Conclusion:**

Paramedics demonstrated a high level of diagnostic accuracy in identifying both the presence and absence of CCA pulsation using two-dimensional (2D) ultrasound across multiple imaging modes in a controlled study setting. The findings suggest that the combination of Color Doppler and, in particular, M−mode appears to be the most suitable approach for identifying CCA pulsation via ultrasound.

## Graphical abstract

**Figure:**
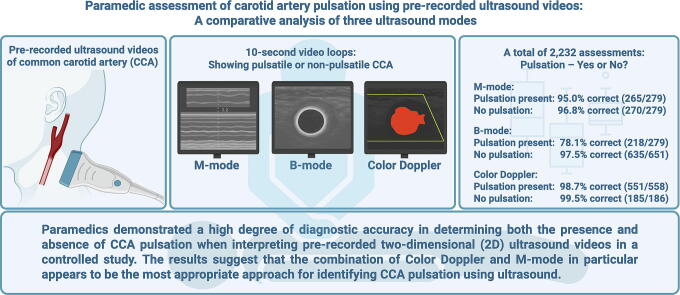

## Introduction

The assessment of pulse and cardiac rhythm is a cornerstone of international cardiopulmonary resuscitation (CPR) guidelines. However, even experienced providers may struggle to detect pulselessness or return of spontaneous circulation (ROSC) by manual palpation, potentially delaying effective chest compressions and critical interventions.^1^ Acknowledging these limitations, the European Resuscitation Council (ERC) advises limiting pulse checks to 10 s.^2^ Manual palpation is inherently subjective, time-consuming, and often unreliable − especially in unconscious patients or during active CPR. As a result, ultrasound has emerged as a valuable tool for assessing pulsatile flow and cardiac activity.^3^ In addition to identifying reversible causes of cardiac arrest, ultrasound can distinguish between absent cardiac activity and severely reduced contractility that fails to produce a palpable pulse − a distinction crucial in pulseless electrical activity (PEA).^4^, ^5^ Pseudo-PEA, a subtype of PEA, presents with organized ECG activity and weak myocardial contractions. These may cause minimal ejection, a faint pulse wave, and visible motion or color flow in the common carotid artery (CCA) on Doppler ultrasound, yet often remain insufficient to generate a palpable pulse. Transthoracic echocardiography (TTE) is commonly used to assess contractility but may be limited during CPR by defibrillator pads, mechanical devices, or poor acoustic windows. In contrast, the CCA is readily accessible, making carotid ultrasound (B-mode, M-mode, or Color Doppler) a rapid, non-invasive method for detecting pulsation during cardiac arrest, particularly in cases of PEA. Prehospital CPR teams include providers with diverse training levels − physicians, paramedics, and EMS personnel. Although diagnostic responsibilities have historically been assigned to physicians, critical situations often necessitate delegation. With broader ultrasound access and training, paramedics increasingly use point-of-care ultrasound (POCUS) in the field. POCUS by non-physician providers is gaining global recognition and has been integrated into paramedic education.^6^, ^7^, ^8^ In a previous study, we showed that medical students with limited ultrasound training could accurately assess carotid pulsation in controlled conditions across several imaging modes.^9^ Building on these findings, we evaluated whether paramedics − key figures in prehospital care − could similarly distinguish between pulsatile and non-pulsatile states using randomized 10-second ultrasound videos of the CCA. To replicate clinical conditions where manual pulse checks often fail − such as post-resuscitation or in pseudo-PEA − we selected two distinct phases of cardiac surgery. These allowed us to simulate reduced pulsatile flow and circulatory standstill, enabling pulse detection to be assessed under conditions that closely reflect real-world prehospital scenarios.

## Methods

### Ethics approval and setting

This cross-sectional study was conducted at the Department of Anesthesiology and Intensive Care Medicine, University Hospital Giessen and Marburg, Campus Marburg, Germany, between 29 May 2024 and 1 December 2024. The study was registered in the German Clinical Trials Register (DRKS00033182, registered on 06 December 2023). Ethics approval was prospectively obtained from the Ethics Committee of the Medical Faculty of Philipps University Marburg (Ref. No. 23/274 ANZ, granted on 09 November 2023). This manuscript adheres to the current Reporting of Observational Studies in Epidemiology (STROBE) guidelines.

### Participants

In the German EMS system, professional designations differ from those commonly used in Anglo-American contexts.^10^ The entry-level certification for non-physician EMS personnel is referred to as EMT-Basic (“Rettungssanitäter”). Further qualifications include the now-outdated EMT-Paramedic (“Rettungsassistent”), which has been replaced by the advanced paramedic (“Notfallsanitäter”) following a comprehensive three-year training program. Some advanced paramedics also hold additional qualifications, such as serving as Helicopter Emergency Medical Service Technical Crew Members (HEMS-TC). For the purpose of this study, and to ensure clarity and consistency, all non-physician EMS professionals are referred to collectively as paramedics, regardless of their individual certification level or specific role.

### Study design

Paramedics in three German counties were invited to participate voluntarily. They were informed about the online study via emails from local rescue service providers, newsletters, and posters at ambulance stations. By scanning a provided QR code, participants accessed the secure study platform, SurveyMonkey (San Mateo, CA, USA).

The landing page contained written information on study procedures and a six-minute introductory video demonstrating correct transducer positioning and briefly introducing the three ultrasound modes (B-mode, M-mode, Color Doppler). Each mode was illustrated with an example video showing the CCA with and without pulsation. Participants then completed an electronic consent form. Upon consent, they viewed 24 pre-recorded anonymized 10-second ultrasound videos (B-mode, M-mode, Color Doppler) presented in randomized order. Each video was played only once and followed by a classification task (pulsation present or absent). The assessment was supplemented by two demographic questions, three on ultrasound experience and perception, three related to CPR, and additional questionnaire items assessing scenario-based CPR decision-making. Web cookies were used to ensure single participation per user and to prevent multiple submissions. The study design closely follows the methodology as our previous work among medical students, including identical video duration, instructional video, presented ultrasound modes, and binary response format (‘pulsation present’/‘no pulsation present’).^9^ However, all 24 ultrasound videos used in the current study were newly generated, with no overlap with the video pool used in the prior study.

### Recording of the ultrasound videos

The ultrasound videos were recorded in advance on patients scheduled for cardiac surgery with cardiopulmonary bypass (CPB). Preoperative diagnostics of the extracranial vessels revealed no relevant abnormalities. All patients provided written informed consent for the acquisition and research use of anonymized ultrasound videos.

To replicate clinical scenarios in which manual pulse checks are unreliable, ultrasound videos of the CCA were recorded during arterial cannulation of the aortic arch under controlled hypotensive conditions. At the time of cannulation, systolic blood pressure was temporarily lowered to 70–80 mmHg according to the clinic’s standard protocol and interdisciplinary consultation. Despite hypotension, arterial pulse waveforms remained detectable via invasive blood pressure monitoring, allowing video acquisition of CCA pulsation at low perfusion pressure.

Non-pulsatile videos were recorded after aortic clamping, during surgical phases in which CPB blood flow was deliberately reduced to zero for more than 10 s, such as during oversewing of vessels. All recordings were anonymized before being presented to participants.

**Fig. 1:**
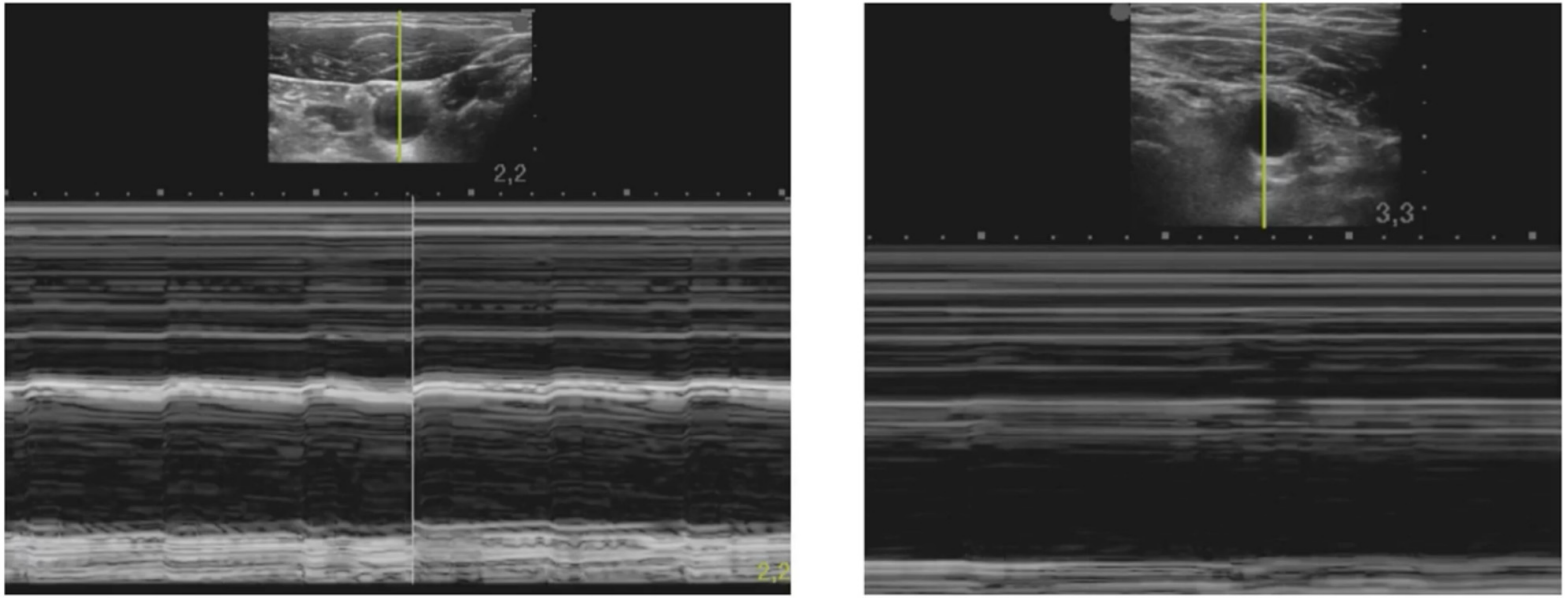
The figure shows an example screenshot from the six-minute introductory video to the study, which was presented to each participant before the study was conducted. It shows an ultrasound examination of the CCA in M−mode. On the left side of the video, a pulsating CCA and on the right side without pulsation (with clamped aorta).

**Fig. 2:**
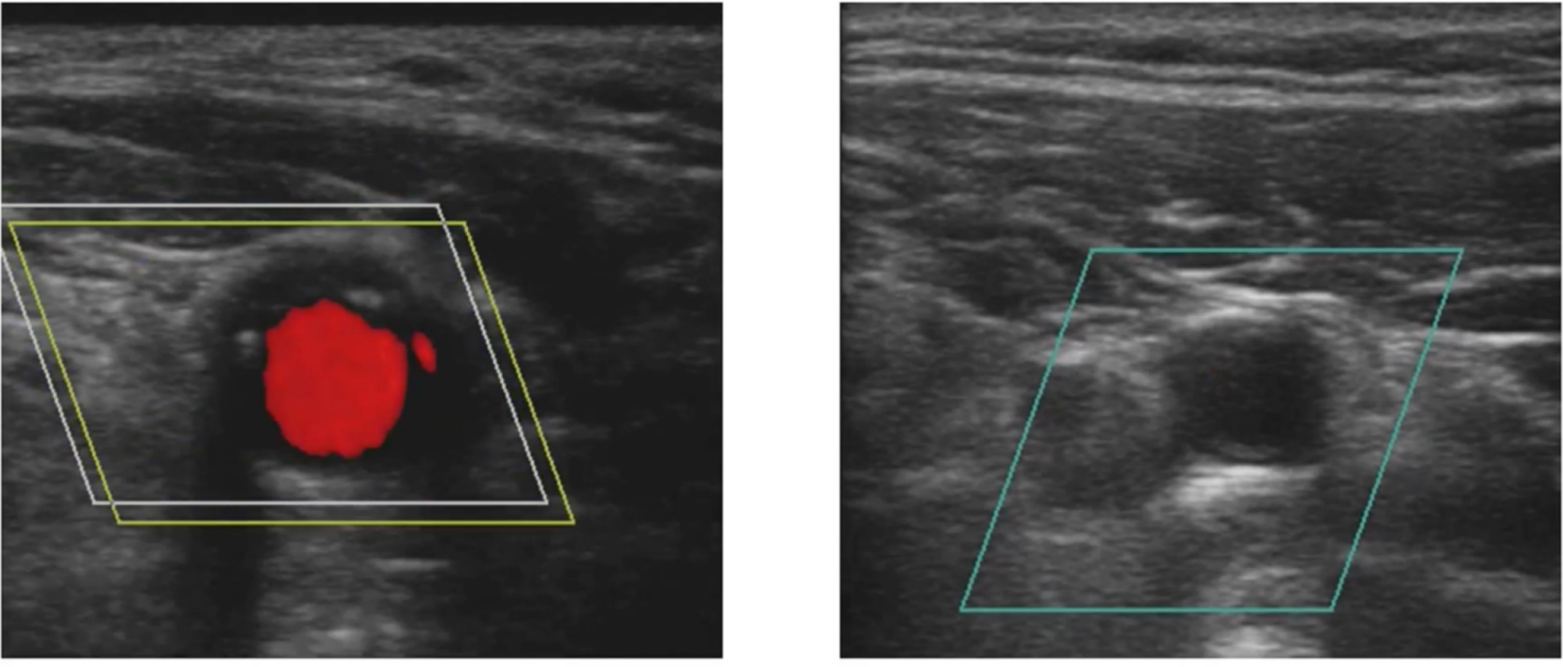
The figure shows an example screenshot from the six-minute introductory video to the study, which was presented to each study participant before the examination was carried out. It shows an ultrasound examination of the CCA in Color Doppler: on the left with a pulsating CCA and on the right without pulsation of the CCA (with clamped aorta).

Ultrasound videos were acquired using the FUJIFILM SonoSite Edge II system (FUJIFILM SonoSite, Bothell, Washington, USA) with the ‘vessel’ preset activated. All videos were recorded by a single investigator, who positioned a linear transducer over the CCA in the short axis, ensuring that the vessel remained centered (see Fig. 1, Fig. 2).

A total of 40 ultrasound videos were generated under standardized conditions using B-mode, M−mode, or Color Doppler, each depicting either a pulsating or non-pulsating CCA. From this pool, 24 videos were randomly selected for the study, ensuring a balanced distribution of 12 videos with CCA pulsation and 12 without. Six M−mode videos were included: three recorded during arterial cannulation (with pulsation) and three after aortic clamping (without). Ten B-mode videos were selected (three with pulsation, seven without), and eight Color Doppler videos (six with pulsation, two without). In all Color Doppler videos, Doppler was activated at the start and centered over the vessel.

### Statistical analysis

Data management and analysis were performed using Excel 2013 (Microsoft; Redmond, Washington, USA). To calculate confidence intervals for sensitivity and specificity within each mode, the clustered data structure was taken into account by applying variance inflation factors (VIFs) as described previously.^9^, ^11^

### Objectives

To the best of our knowledge, this study is the first to investigate whether paramedics were able to correctly discriminate between ‘pulsation present’ and ‘no pulsation present’ using B-mode, M−mode, or Color Doppler ultrasound videos of the CCA. Further endpoints included exploring associations with demographic characteristics of the paramedics, including previous professional experience and theoretical as well as practical expertise in ultrasound.

## Results

A total of 116 paramedics took part in this online study. Of these, 93 completed the study in full, enabling a total of 2232 ‘pulsation present’ or ‘no pulsation present’ decisions to be evaluated.

### Professional experience and training level

Of the participants, 20 % (19/93) reported having worked in the emergency medical services for less than five years, 49 % (46/93) for five to ten years, 17 % (16/93) for 11 to 20 years, and 13 % (12/93) for more than 20 years. Participants were also asked about their highest completed qualification (ranked in order from lowest to highest level). Specifically, 24 % (22/93) held the entry-level EMT-Basic (“Rettungssanitäter”), 2 % (2/93) were trained as EMT-paramedics (“Rettungsassistent”), 72 % (67/93) were certified as advanced paramedics (“Notfallsanitäter”), and 2 % (2/93) served as helicopter emergency medical service technical crew members (HEMS-TC).

### Experience in the field of ultrasound

The participants were asked to what extent basic ultrasound principles had been covered during their previous training. 59 % (55/93) reported that they had received both theoretical and practical instruction (including hands-on training on patients or volunteers), 24 % (22/93) indicated that only theoretical aspects had been taught, while 17 % (16/93) stated that they had not received any ultrasound-related education at all.

In addition, participants were asked to rate the following statement: ‘I currently feel sufficiently confident in performing ultrasound examinations/ videos of the carotid artery’. 23 % (21/93) agreed with the statement, and 6 % (6/93) strongly agreed. 15 % (14/93) neither agreed nor disagreed, while 47 % (44/93) disagreed and 8 % (7/93) strongly disagreed. 1 % (1/93) responded with ‘I don’t know’.

### Ultrasound during CPR

Furthermore, participants were asked to rate the statement ‘The use of ultrasound in the context of prehospital CPR is useful’ using a Likert scale. In total, 87 % (81/93) of participants agreed with the statement, with 30 % (28/93) strongly agreeing and 57 % (53/93) agreeing. 12 % (11/93) were undecided (neither agree nor disagree), and 1 % (1/93) strongly disagreed. No participants chose ‘disagree’ or ‘I don’t know’.

### Experience in the field of CPR

The majority of participants (99 %, 92/93) reported having already performed CPR on a person. Only 1 % (1/93) stated that they had never performed CPR. Participants were also asked how often they had attended CPR training. 56 % (52/93) reported participating in resuscitation training several times a year, while 40 % (37/93) stated that they attend training once a year. 1 % (1/93) indicated participation every two years and 3 % (3/93) stated they train less frequently than every two years.

### Scenario for pulse check during CPR

The participants were presented with the following scenario: During ongoing prehospital CPR, a rhythm and pulse check is required. The ECG shows regular electrical activity. An ultrasound device is available on scene. Participants were asked how they would assess whether electrical activity is associated with a mechanical pulse. 74 % (69/93) stated that they would manually check the pulse, while 26 % (24/93) reported that they would use ultrasound to assess for the presence or absence of a pulse.

Those who indicated that they would rely on manual pulse check (75 %, 69/93) were further asked to specify their reasoning. None of the participants expressed general mistrust in ultrasound for pulse checks. However, 75 % (52/69) stated that they would use ultrasound for pulse checks once they feel confident in the technique. The remaining 25 % (17/69) selected the response ‘I consider ultrasound useful for pulse checks, but the effort involved is currently too great’.

In the previously described scenario (electrical activity visible on the ECG during an ongoing prehospital CPR), participants were asked which vessel they would currently use to perform a manual pulse check. The majority of participants (80 %, 74/93) indicated the carotid artery, followed by 18 % (17/93) who choose the femoral artery, and 2 % (2/93) who reported the radial artery. No participant selected the subclavian artery for manual pulse check.

In addition, the participants were asked which vessel they would use for ultrasound-assisted pulse check. 80 % (74/93) selected the carotid artery, 15 % (14/93) reported that they would use TTE, 4 % (4/93) the subclavian artery, and 1 % (1/93) the femoral artery. No participant indicated the radial artery as the preferred site for ultrasound assessment.

Participants were then asked to assess the therapeutic consequence of a confirmed arterial pulse on ultrasound in the described scenario: in addition to electrical activity on the ECG, vascular pulsation of the CCA is observed during the rhythm and pulse check via ultrasound, but no pulse is palpable.

39 % (36/93) of participants considered continued chest compressions to be appropriate in this situation, whereas 61 % (57/93) deemed further compressions to be inappropriate.

### M-mode

A total of six ultrasound videos in M−mode were presented. The numbers given below the percentages always refer to the number of decisions to be made. In cases where CCA pulsation was present in M−mode the correct response ‘CCA pulsation present’ was selected in 95 % (265/279) of cases. In contrast, in 5 % (14/279) of cases, participants incorrectly selected ‘CCA pulsation not present’.

For M−mode videos without visible pulsation of the CCA, the option ‘CCA pulsation not present’ was correctly chosen in 97 % (270/279) of cases. However, in 3 % (9/279) of cases, ‘CCA pulsation present’ was selected even though no pulsation was present (see Table 1).

**Table 1:** The table shows the distribution of the decisions ‘pulsation present’ and ‘no pulsation present’ when videos in M−mode with or without a pulse (and clamped aorta) were presented. CCA = Common Carotid Artery.

M−mode	CCA pulsation present	CCA pulsation not present	Total
Decision “CCA pulsation present”	265	9	274
Decision “CCA pulsation not present”	14	270	284
Total decisions	279	279	

### B-mode

Within the three videos in B-mode and pulsation of the CCA, the decision ‘pulsation present’ was made in 78 % (218/279) of the cases. In contrast, ‘no pulsation present’ was selected in the remaining 22 % (61/279), although the video showed pulsation of the CCA. In the seven B-mode videos without pulsatile CCA, the option ‘no pulsation present’ was selected in 98 % (635/651) of the cases. By contrast, the option ‘pulsation present’ was selected in 2 % (16/651) of the responses, even though no pulsation was present (see Table 2).

**Table 2:** The table shows the distribution of the decisions ‘pulsation present’ and ‘no pulsation present’ when videos in B-mode with or without a pulse (and clamped aorta) were presented. CCA = Common Carotid Artery.

B-mode	CCA pulsation present	CCA pulsation not present	Total
Decision “CCA pulsation present”	218	16	234
Decision “CCA pulsation not present”	61	635	696
Total decisions	279	651	

### Color Doppler

In the six videos with activated Color Doppler and pulsation of the CCA, the decision ‘pulsation present’ was selected in 99 % (551/558) of the videos shown. In the remaining 1 % (7/558) of cases, ‘no pulsation present’ was selected, although the video showed CCA pulsation with activated Color Doppler. Within the two videos with Color Doppler without pulsating CCA, 99 % (185/186) of the videos were assessed as ‘no pulsation present’. In contrast, the option ‘pulsation present’ was selected in 1 % (1/186) of the cases, even though no pulsation was present (see Table 3).

**Table 3:** The table shows the distribution of the decisions ‘pulsation present’ and ‘no pulsation present’ when videos in Color Doppler mode with or without a pulse (and clamped aorta) were presented. CCA = Common Carotid Artery.

Color Doppler	CCA pulsation present	CCA pulsation not present	Total
Decision “CCA pulsation present”	551	1	552
Decision “CCA pulsation not present”	7	185	192
Total decisions	558	186	

**Fig. 3:**
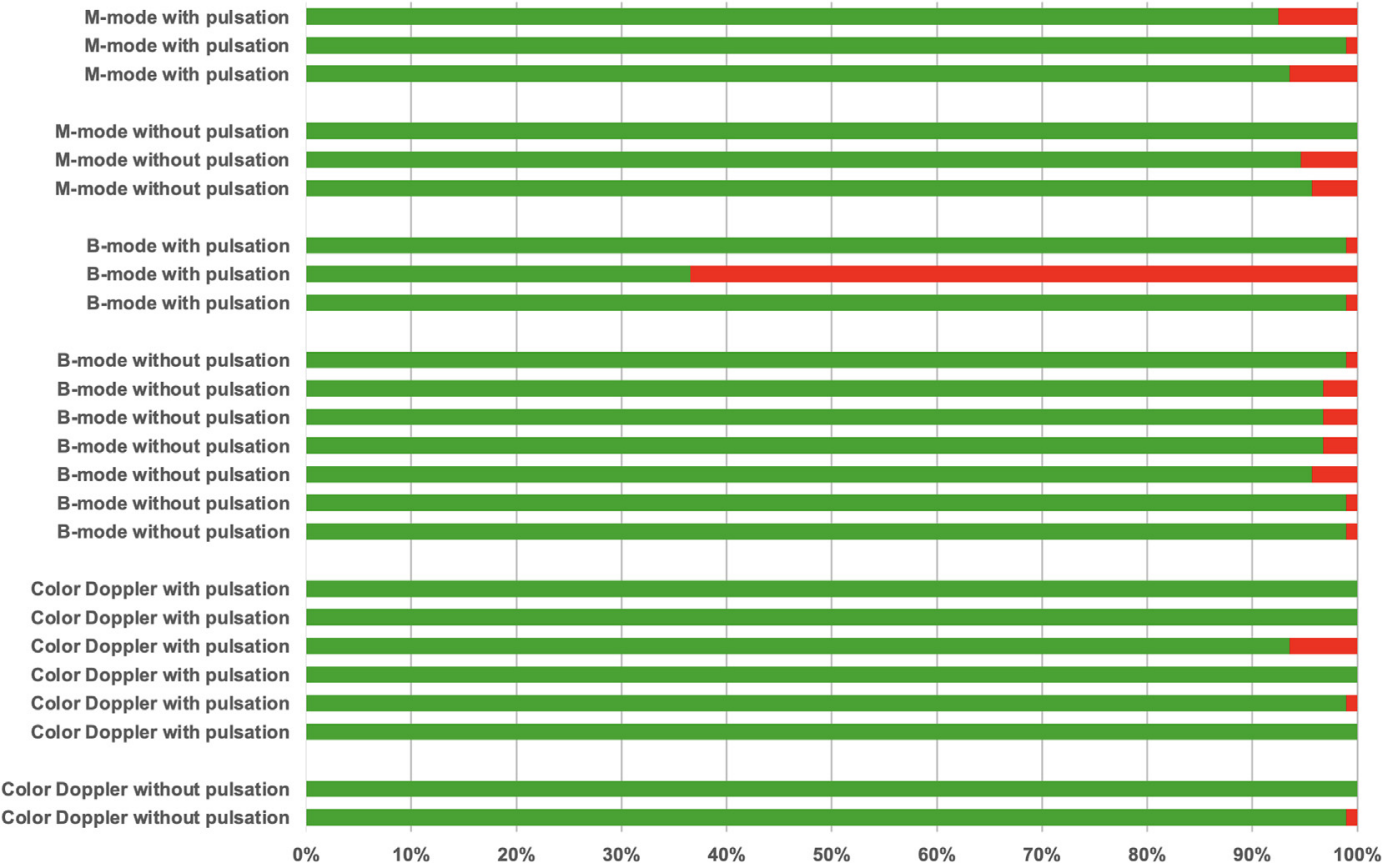
Distribution of correct and incorrect classifications for all 24 ultrasound videos, totaling 2232 individual decisions. Each row represents one video, grouped by ultrasound mode (M−mode, B-mode, Color Doppler) and by the presence or absence of pulsation. Green bars indicate the proportion of participants who classified the video correctly; red bars indicate the proportion who classified it incorrectly. (For interpretation of the references to color in this figure legend, the reader is referred to the web version of this article.)

The sensitivity, specificity, and corresponding confidence intervals for the three ultrasound modes are presented in Table 4. For the binary diagnostic decision of ‘pulsation present’ versus ‘no pulsation present’, Color Doppler videos demonstrated the highest sensitivity and specificity. The distribution of the of 2232 decisions made by the participants in evaluating the videos is shown in Fig. 3.

**Table 4:** Diagnostic accuracy (sensitivity and specificity) for detecting CCA pulsation across ultrasound modes, with 95% confidence intervals.

	M−mode	B-mode	Color Doppler
Sensitivity (95 % CI)	94.98 % (74.43–100 %)	78.14 % (39.23–100 %)	98.75 % (91.34–100 %)
Specifity (95 % CI)	96.77 % (94.52–99.03 %)	97.54 % (96.25–98.84 %)	99.46 % (98.32–100 %)

### Self-assessment

Finally, the participants were asked to answer the following question using a Likert scale: ‘After participating in this study, I am now more confident that I can reliably determine whether or not pulsation is present based on ten-second ultrasound videos of the CCA.’

26 % (24/93) of the participants ‘strongly agreed’, while 41 % (38/93) ‘agreed’ with the statement. A total of 13 % (12/93) chose the option ‘neither agree nor disagree’, 14 % (13/93) ‘disagreed’ with the statement, and 4 % (4/93) ‘strongly disagreed’. 2 % (2/93) were unsure or declined to answer.

## Discussion

Assessing hemodynamic status during CPR remains particularly challenging in the prehospital setting, where manual pulse checks are frequently unreliable. POCUS of the CCA offers a potential diagnostic alternative. In this study, we demonstrated that paramedics could accurately identify between ‘pulsation present’ and ‘no pulsation present’ by interpreting 2D ultrasound videos of the CCA. Among the evaluated ultrasound modes (M−mode, B-mode, Color Doppler), Color Doppler showed the highest sensitivity and specificity for detecting CCA pulsation. These findings suggest that ultrasound may serve as a valuable tool for objective pulse verification during CPR, thereby improving the reliability of pulse checks.

Prehospital ultrasound has gained substantial importance in recent years and is incorporated into international CPR guidelines.^4^, ^12^ Despite ongoing technological advancements − including the development of compact and portable ultrasound devices − its global prehospital availability remains heterogeneous, and comprehensive data for Europe and other regions are lacking. Regional studies suggest a trend toward increasing adoption: in North America, prehospital ultrasound remains infrequent but is growing; in Europe, availability varies widely, with reported rates of 52 % in Germany, 69 % in Austria and a steady increase in France since 2011.^13^, ^14^, ^15^, ^16^, ^17^, ^18^

In Germany, prehospital ultrasound is mainly performed by physicians, with a key barrier to its use by paramedics being the absence of targeted training programs and curricula.^19^ In contrast, international studies have shown that structured prehospital ultrasound training for paramedics is feasible and clinically useful, particularly for cardiac and pulmonary assessments.^19^, ^20^, ^21^, ^22^, ^23^ Despite these findings, routine implementation by paramedics outside research settings is still rarely implemented.

In our study, many participants reported basic theoretical or practical exposure to ultrasound, likely acquired during in-hospital training, particularly within anesthesiology or the emergency department, where ultrasound is routinely used. In addition, the study region offers widespread access to prehospital ultrasound devices, which may have contributed to general familiarity. However, more than half of the participants in this study ‘(strongly) disagreed’ with the statement, ‘I currently feel sufficiently confident in performing ultrasound videos of the CCA’. This self-assessment referred to participants’ general prior experience and confidence in conducting ultrasound examinations of the CCA − not to the recordings used in this study, which focused solely on the interpretation of pre-recorded ultrasound videos. The reported lack of confidence likely reflects the absence of CCA ultrasound in paramedic education. Further supporting this, many participants indicated they would consider using ultrasound for pulse checks if more comprehensive training were available.

Previous studies have demonstrated the feasibility of CCA ultrasound for non-invasive, real-time hemodynamic monitoring, as well as the feasibility of structured ultrasound use by paramedics during prehospital CPR.^24^, ^25^, ^26^, ^27^ Furthermore, paramedics can acquire ultrasound competencies within a few hours to days, with significant improvements in pre- and post-training performance.^19^, ^20^, ^21^, ^28^, ^29^ Similar results have been reported specifically for CCA pulsation assessment.^1^, ^27^ Even theoretical instruction alone has been associated with increased confidence in ultrasound video interpretation.^3^, ^9^, ^30^ In this context, digital teaching formats offer a resource-efficient, scalable approach to enhance ultrasound competence among prehospital providers. Consistent with these findings, nearly 70 % of participants in our study reported increased confidence in identifying CCA pulsations after completing the study. The brief instructional video provided at the beginning appears to have contributed substantially to this improvement, highlighting that even low-effort interventions can yield meaningful educational effects.

For both manual and ultrasound-guided assessments, the CCA was the preferred anatomical site among participants. This preference likely reflects the CCA’s easily accessible location, especially for providers positioned at the head of the patient to manage airway and ventilation during CPR. From this position, the artery can be assessed during rhythm checks without interrupting team coordination. Moreover, maintaining the ultrasound probe on the neck does not interfere with chest compressions and minimizes interruptions.^31^ While ultrasound detection of cardiac activity via POCUS may prolong hands-off times during CPR beyond the recommended 10-second limit, the detection of pulsation via vessel compression or Doppler imaging may facilitate faster assessments.^32^ In a case series, Simard et al. showed that ultrasound-guided compression of carotid or femoral vessels could reliably confirm the presence or absence of pulsations within five seconds, even in cases with non-palpable pulse or inconclusive clinical findings.^31^

Although existing data suggest that CCA pulse detection via ultrasound can be completed in under 10 s, appropriate selection of ultrasound modes remains critical for reliable interpretation.^33^ Koch et al. demonstrated that Color Doppler can be used to quantify CCA blood flow in the context of ROSC, indicating its potential for both pulse detection and assessment of flow dynamics.^24^ Supporting this, Germanoska et al. reported that Color Doppler may detect pulsatile flow in the CCA earlier and at lower mean arterial pressures than B-mode imaging, although diagnostic reliability remains limited.^34^

In our study, videos using Color Doppler demonstrated the highest sensitivity and specificity in detecting CCA pulsations. Although B-mode is considered the most basic, user-friendly ultrasound mode and does not require additional device settings, it was the least accurate in correctly identifying CCA pulsations in our study. In contrast, Kang et al. previously demonstrated that physicians using B-mode were able to detect a pulse in less than 10 s after ROSC, even after a short training period, and did so more rapidly than with manual palpation.^35^ Therefore, B-mode may still represent a useful tool for paramedics during CPR, as our study found it more accurate than M−mode in correctly identifying the absence of a pulse.

The unexpectedly favorable diagnostic performance of M−mode – especially in detecting CCA pulsations – was notable, given that motion artifacts, such as those resulting from inconsistent probe handling, can be misinterpreted over time as vascular wall motion, thereby complicating the distinction between true pulsations and artifacts.^36^

Although our study focused on paramedics, carotid ultrasound has also been investigated in other clinical settings, including by physicians and nurses in emergency departments.^24^, ^25^, ^33^, ^34^, ^35^ This reflects the growing interdisciplinary interest in carotid ultrasound as a non-invasive supplement to manual pulse measurement in cardiac arrest. More advanced techniques such as tissue Doppler imaging may also be of interest to detect not only pulsation but also flow within the carotid artery.^36^ Monitoring carotid artery flow could enable timely and effective detection of ROSC, particularly in cases of pseudo-PEA or low-flow states.^37^ Further studies are needed to investigate the applicability of these techniques at different levels of care and in different clinical settings.

Pseudo-PEA represents severe cardiogenic shock rather than true arrest, with a more favorable prognosis if promptly recognized.^38^ Although our study did not focus on modality-specific detection of pseudo-PEA, over half of the participants stated they would terminate chest compressions upon visualizing CCA pulsations despite absence of a palpable pulse. According to current AHA guidelines, ROSC may be assumed if at least one of the following is present: a palpable pulse or measurable blood pressure, a sustained rise in PETCO_2_ (≥40 mmHg), or spontaneous arterial pressure waves.^39^ In cases of pseudo-PEA, where arterial pressure may be insufficient for manual detection, the combination of organized ECG activity, PETCO_2_ increase, and visible CCA pulsation could support the decision to pause CPR. Although this approach is not yet part of the formal criteria, further studies could support the feasibility of this combination. Patients with pseudo-PEA may respond to simple interventions such as fluid resuscitation, inotropic support or treatment of reversible causes.^31^, ^40^ Echocardiographic identification of pseudo-PEA followed by vasopressor administration and cessation of chest compressions has been associated with higher rates of ROSC, survival to discharge, and favorable neurological outcomes.^41^ Similarly, Simard et al. described successful outcomes in pseudo-PEA using ultrasound-guided arterial compression and low-dose vasopressors without further CPR.^31^ Additionally, findings from the PARAMEDIC-2 trial showed that vasopressor use, particularly epinephrine, was associated with increased risk of severe neurological impairment, supporting their use only in confirmed cardiac arrest without evidence of perfusion.^42^ This underscores the importance of accurately distinguishing between true and pseudo-PEA. Our findings suggest that ultrasound assessment of the CCA may serve as a valuable adjunct in distinguishing pseudo-PEA from true PEA during CPR.

Ultrasound guidance may also offer psychological support to EMS personnel during CPR. Studies show increased clinician confidence in terminating resuscitation when cardiac standstill is confirmed on both ECG and ultrasound.^40^ Incorporating standardized ultrasound criteria − such as absent contractility or vascular pulsation − into CPR termination protocols may improve decision-making and reduce emotional burden in the prehospital setting.^40^, ^43^

### Strengths and limitations of the study

A key strength of this study is the large number of evaluated decisions – 2232 in total. In contrast to previous studies, which have primarily focused on the interpretation of POCUS videos of the heart, lungs, and abdomen, our study specifically examined the interpretation of CCA ultrasound videos by paramedics. It is known that CCA pulsation becomes increasingly difficult to detect at lower systolic blood pressure levels, with sensitivity decreasing accordingly.^3^ To better reflect clinical conditions associated with hemodynamic compromise, the videos used in this study showed CCA pulsation at systolic blood pressure levels of approximately 70–80 mmHg. Another strength is the inclusion of paramedics as the primary study population – rather than physicians or in-hospital emergency personnel, as seen in most previous studies.^3^, ^30^ This approach addresses a highly relevant and growing professional group in the context of expanding prehospital care competencies. Additionally, our study compared three different ultrasound modes (B-mode, M−mode, and Color Doppler), offering a broader evaluation of their respective diagnostic value for detecting CCA pulsation – whereas prior investigations typically focused on only one or two ultrasound modes.^3^, ^30^, ^34^

However, several limitations must be acknowledged when interpreting our findings.

First, our study assessed participants’ ability to interpret pre-recorded ultrasound videos, rather than the acquisition process itself. The ability to obtain and optimize acoustic windows − especially under time pressure during CPR − was not assessed in this study. This represents a relevant limitation of our study, as image acquisition skills may vary depending on the training background and experience of the provider. While previous studies have shown that paramedics can obtain diagnostic-quality images after focused instruction, image quality in the field may still vary.^22^, ^44^, ^45^ Differences in training duration, participant background, and ultrasound protocols across studies further complicate generalizability.^6^, ^46^ Future research should address both image acquisition and interpretation in real-life prehospital settings to ensure reproducible performance.

Second, the study was conducted in a controlled online environment, without the time-critical and stressful conditions typical of real-life cardiac arrest scenarios, which may affect diagnostic performance.

Third, all ultrasound videos were recorded by a single experienced investigator. While this ensured videos consistency and minimized artefacts, it may limit generalizability to routine practice with less experienced users or inferior ultrasound video quality.

Fourth, individual videos may have disproportionately influenced diagnostic outcomes, as only a small number of cases per mode and classification group were included. The relatively low sensitivity of 78.14 % in B-mode may be attributable to a single video that was frequently misclassified as ‘no pulsation’ despite visible pulsation (see Fig. 3). A study design including more videos and fewer participants may yield more representative results and reduce the influence of outliers.

Fifth, the distribution across ultrasound modes was unequal due to random selection from a larger video pool without allocation. Although this imbalance was not intentional, future studies should apply stratified randomization to ensure balanced representation of modalities and enable more robust comparisons.

Sixth, voluntary participation may have introduced a potential selection bias in the study sample. It is possible that individuals with no ultrasound knowledge chose not to participate, while those with some relevant experience were overrepresented. This may have influenced the sensitivity and specificity estimates.

Additionally, limiting the number of videos per participant was an intentional methodological trade-off to reduce cognitive fatigue and maximize completion rates. While this approach likely improved data quality, it may have limited participants’ exposure to diagnostic variability across ultrasound modes.

## Conclusion

This study analyzed 2232 assessments by paramedics regarding the presence or absence of CCA pulsation in pre-recorded 10-second ultrasound videos. Paramedics demonstrated high diagnostic accuracy across imaging modes, with Color Doppler and M−mode performing best in detecting pulsation. These findings suggest that CCA ultrasound may serve as a reliable adjunct to manual pulse checks during CPR, especially in hypotensive states where traditional assessment is challenging. Further clinical studies are warranted to evaluate its applicability under real-world conditions.

## Data availability

The datasets generated and/or analysed during the current study are available from the corresponding author on reasonable request.

## CRediT authorship contribution statement

**C. Gaik:** Writing – review & editing, Writing – original draft, Methodology, Formal analysis, Conceptualization. **H. Wulf:** Writing – review & editing. **B. Vojnar:** Writing – review & editing, Writing – original draft, Project administration, Methodology, Conceptualization.

## Ethics approval

Ethics approval was obtained from the Ethics Committee of the Medical Faculty of 10.13039/100008967Philipps University Marburg (Ref. No. 23/274 ANZ, approved on 09 November 2023). All participants provided written informed consent prior to study participation.

## Funding

No funding was received for this study.

## Declaration of competing interest

The authors declare that they have no known competing financial interests or personal relationships that could have appeared to influence the work reported in this paper.
